# Isolation and screening of phosphorus solubilizing bacteria from saline alkali soil and their potential for Pb pollution remediation

**DOI:** 10.3389/fbioe.2023.1134310

**Published:** 2023-02-06

**Authors:** Chaonan Zhang, Haoming Chen, Yao Dai, Yan Chen, Yuxin Tian, Zongli Huo

**Affiliations:** ^1^ School of Environmental and Biological Engineering, Nanjing University of Science and Technology, Nanjing, China; ^2^ Jiangsu Provincial Center for Disease Control and Prevention, Nanjing, China

**Keywords:** phosphorus solubilizing bacteria, salt and alkaline tolerant, Pb stress, organic acid, extracellular polymers, mineralization reaction

## Abstract

The high pH and salinity of saline alkali soil not only seriously restrict the growth of crops, but also aggravate the pollution of heavy metals. The fixation of heavy metals and the regulation of pH by phosphorus solubilizing microorganisms may become a new way to repair heavy mental and improve saline alkali soil. In this study, a saline-alkali resistant bacteria (CZ-B1, CGMCC No: 1.19458) was screened from saline-alkali soil, and its tolerance to salt/alkali/lead stress was investigated by shaking flask experiment. The strain was identified as *Bacillus amyloliquefaciens* by morphology and 16S rRNA gene sequence analysis. The optimum growth temperature of CZ-B1 is about 35°C–40℃. The maximum salt stress and pH that it can tolerance are 100 g/L and 9 respectively, and its tolerance to Pb^2+^ can reach 2000 mg/L. The phosphorus release amount of CZ-B1 to Ca_3_(PO_4_)_2_ within 72 h is 91.00–102.73 mg/L. The phosphate solubilizing index in PVK agar medium and NBRIP agar medium are more than 2, which can be defined as phosphate solubilizing bacteria. Moreover, the dissolution of CZ-B1 to phosphorus is mainly attributed to tartaric acid, citric acid and succinic acid in inorganic medium. In addition, the removal rate of Pb^2+^ by CZ-B1 can reach 90.38% for 500 mg/L. This study found that CZ-B1 can immobilize Pb through three biological mechanisms (organic acid, extracellular polymers and mineralization reaction). The release of succinic acid (10.97 g/L) and citric acid (5.26 g/L) may be the main mechanism to promote the mineralization reaction of CZ-B1 (phosphate and oxalate) and resistance to Pb stress. In addition, the high enrichment of Pb^2+^ by EPS can increase the rate of extracellular electron transfer and accelerate the mineralization of CZ-B1. The screening and domestication of saline-tolerant phosphorus-solubilizing bacteria not only help to remediate Pb contamination in saline soils, but also can provide P element for plant growth in saline soil.

## Highlights

• A strain of phosphorus solubilizing bacteria (CZ-B1) tolerant to salt and alkaline stress was successfully screened.
• The CZ-B1 tolerated Pb^2+^, salinity and alkalinity up to 2000 mg/L, 10% and 9.
• The resistance of CZ-B1 to Pb is mainly achieved by cellular and secretions adsorption under low Pb stress.
• CZ-B1 can form pyromorphite and Pb oxalate minerals through secreted citric, succinic and oxalic acids.
• CZ-B1 increases extracellular electron transfer rate and mineral precipitation efficiency by EPS adsorption and enrichment of Pb^2+^.

## 1 Introduction

Soil salinization has always been a major threat to the sustainable development of agriculture and the improvement of land use efficiency (Meena et al., 2020; Liang et al., 2021; Chen Q. et al., 2022). It is reported that more than 100 million hectares of land in the world are being destroyed by soil salinization (Wicke et al., 2011), and the area of saline alkali soil continues to grow at a rate of 10% every year (Cui et al., 2021). There are over 20,000 km^2^ of saline soils in China’s coastal mudflats that need to be improved (Cui et al., 2021). The high salinity and high pH of saline alkali soil will lead to severe degradation of soil structure, nutrient deficiencies and disruption of microbial activity, among other hazards (He et al., 2020; Zhao et al., 2020; Zhang et al., 2022), which disrupts the normal physiological functions of plant cells (Shao et al., 2018; Jia et al., 2019). Not only that, but high salinity of saline alkali soil will affect the diffusion of heavy metals (HMs) ions, thus increasing the bioavailability of HMs in soil and their toxicity to plants (Liu et al., 2019). Therefore, in the context of the global food crisis, as an important reserve of land resources for food production and ecological environment construction, the improvement and utilization of salinized soil is imminent (Zhao et al., 2020; Zhang et al., 2022).

As a common HMs pollution, lead (Pb) has high toxicity, bioaccumulation and persistence (Jing et al., 2004; Gladysz-Plaska et al., 2017). Pb can be accumulated and amplified in the organism through the food chain. Excessive Pb intake will seriously affect the human nerve center, hematopoietic function, and cause cancer and other diseases (Kolodynska et al., 2018; Chen H. M. et al., 2022; Lai et al., 2022). Under neutral or alkaline conditions, Pb^2+^ is not active in the soil due to the formation of Pb(OH)_2_ precipitation (Wang et al., 2015). This lead to a large accumulation of Pb^2+^ in saline alkali soil (tilth). However, Pb(OH)_2_ has poor stability and can be dissolved by acid substances secreted by plant roots or soil microorganisms, which has great potential risks (Reboreda and Cacador, 2007). It has been proved that the activity of Pb^2+^ in saline alkali soil will increased after reclamation, which leads to Pb in rice approaching the food safety threshold (Wang et al., 2015). Common remediation technologies for HMs pollution include physical and chemical methods, such as soil cleaning, heat treatment (Phieler et al., 2014), electric remediation (Gupta and Kumar, 2017), stable solidification method (Li et al., 2021) and chemical elution (Leyval et al., 1997). These methods have the disadvantages of high cost, low remediation capacity and secondary contamination (Glick, 2010). Also, these methods are difficult to apply to the remediation of HMs in saline soils (Lefevre et al., 2009). Therefore, a low-cost, sustainable and ecologically friendly measure is urgently needed to alleviate soil salinization stress and improve plant productivity and safety of saline soil.

In recent years, the use of microorganisms for soil remediation and improvement has proved to be a safe and economic potential measure (Sheng et al., 2008). Phosphorus solubilizing microorganisms (PSM), as a type of environmental functional microorganism, can enhance phosphorus utilization by plants via releasing organic acids, enzymes and other biological secretions to dissolve insoluble phosphorus sources in the environment (Rodriguez et al., 2006; Shao et al., 2021; Zhu et al., 2022). In addition, with the further study of PSM, it has been proved that they can be used in the remediation of HMs in soil (Park et al., 2011; Teng et al., 2019; Feng et al., 2022; Lai et al., 2022). The greatest advantage of PSM in HMs remediation is their ability to release phosphate ions that combine with HMs ions to form more stable metal-phosphate compounds (Chen et al., 2020; Lai et al., 2022). For example, under Pb^2+^ stress of 1000 mg/L, phosphorus solubilizing bacteria (*Enterobacter* sp.) can repair Pb pollution of 114.2–231.4 mg/L by forming Pb phosphate precipitation (Chen et al., 2019). The percentage amount of Pb immobilized in solution after 14 days was 98.18% for the PSM (*Citrobacter farmer* CFI-01) (Li et al., 2022). However, the saline alkali environment has a significant inhibitory effect on the growth of microorganisms. High salt content will cause the microbial cells to shrink and lose their activity due to dehydration (Michel et al., 2022). Meanwhile, high concentrations of Pb^2+^ significantly inhibit the growth of phosphorus-dissolving bacteria. For example, PSM (*Enterobacter* sp.) can only survive at Pb^2+^ concentrations of <500 mg/L (Jiang et al., 2020). Therefore, if PSM can be screened from saline alkali soil and their Pb tolerance can be domesticated, it may become a new way to effectively passivate Pb in saline alkali environment.

This study aims to screen a phosphorus solubilizing bacteria that can repair HMs Pb in saline alkali environment, and verify the tolerance of the strain to salt/alkali/Pb stress. At the same time, X-ray diffraction (XRD), infrared spectroscopy (ATR-IR), three-dimensional fluorescence (3D-EEM) and scanning electron microscopy (SEM/EDX) were used to explore the biological mechanism of Pb passivation and repair. It is expected to provide a new idea for remediation of Pb pollution in saline alkali soil.

## 2 Materials and methods

### 2.1 Isolation of salt alkali and Pb resistance bacteria

Soil samples were collected from coastal saline alkali soil in Dongying City, Shandong Province (118.78112°E, 37.83379°N). 10 g soil was added to 100 mL sterilized sterile water containing Pb^2+^ stress for shaking (Pb^2+^ concentration was 50 mg/L, 180 r/min, 30℃, 2 h). Then, the supernatant was continuously diluted and coated on Luria Bertani (LB) solid medium, and incubated in a 36℃ constant temperature incubator for 72 h. The strains with Pb tolerance and salt alkali tolerance can be obtained by picking single colonies of different forms for purification.

### 2.2 Identification of salt alkali and Pb resistance bacteria

A salt alkali resistant strain screened was labeled CZ-B1, and was incubated in LB medium for 72 h (180 rpm, 30℃) by shaking. Then the strain was identified by 16 S rRNA gene sequence. Use FastDNA SPIN Kit (Bio Teke, Co., China) to extract genomic DNA (Zhou et al., 2018). Primers 27F (5′- AGA GTTTGATCMTGCTCAG-3′) and 1492R (5′- CRGYTACCTGTTACGA-3′) were used for PCR amplification of 16S rRNA gene (Biswas et al., 2018).

### 2.3 Phosphorus dissolving capacity experiments

In this study, two different methods were used to verify the phosphorus dissolving function of CZ-B1, namely, the plate phosphorus dissolving method and the liquid medium phosphorus dissolving method. The plate phosphorus dissolving method is to inoculate CZ-B1 onto PVK (Pikovskaya) solid medium and NBRIP (National Botanical Research Institute’s Phosphate) solid medium for 4–7 days, and record the size of its halo (Liu et al., 2015). The method of dissolving phosphorus in liquid medium is to culture CZ-B1 in modified PVK, PVK and NBRIP liquid medium by shaking for 72 h (180 rpm, 30℃). The mixed solution was centrifuged and filtered, and the total soluble phosphorus content in the supernatant was determined (after deducting the control). The PVK medium contains glucose 10 g, (NH_4_)_2_SO_4_ 0.5 g, NaCl 0.3 g, KCl 0.3 g, FeSO_4_.7H_2_O 0.3 g, MgSO_4_.7H_2_O 0.3 g, MnSO_4_.4H_2_O 0.03 g, yeast powder 0.5 g, Ca_3_(PO_4_)_2_ 5 g, H_2_O 1 L, solid medium contains 2% agar (Li et al., 2018). Meanwhile, modified PVK medium (HAP) was obtained by replacing Ca_3_(PO_4_)_2_ in PVK medium with hydroxyapatite (insoluble). NBRIP medium consisted of glucose 10 g, Ca_3_(PO_4_)_2_ 5 g, MgCl_2_.6H_2_O 5 g, MgSO_4_.7H_2_O 0.25 g, KCl 0.2 g, (NH_4_)_2_SO_4_ 0.1 g, H_2_O 1 L, solid medium contains 2% agar (Park et al., 2011).

### 2.4 Stress experiments

The stress culture of CZ-B1 was carried out at different temperatures, pH, salt concentration and Pb^2+^ concentration (Pb(NO_3_)_2_) (Table 1). The absorbance (wavelength 600 nm) of CZ-B1 at different times (1–72 h) was measured separately to characterize the growth of the strain, no stress treatment was used as control. The samples of Pb stress experiment with different concentrations (72 h) were divided into solid and liquid fractions. One part of the liquid samples are used to determine the content of organic acids (formic acid, oxalic acid, malic acid, succinic acid, tartaric acid, and citric acid), 3D-EEM, electrochemical characteristics and EPS. The other part of the liquid samples were centrifuged (5000 rpm, 5 min) and solid-liquid separated, the Pb content in the filtrate was determined by ICP-OES. One part of the solid sample is used to determine ATR-IR, XRD, and the other part is fixed with 2.5% glutaraldehyde for 24 h, then dehydrated with ethanol gradient and used for scanning electron microscopy analysis (Su et al., 2019).

**TABLE 1:** The culture conditions of stress experiment.

Condition	Value
temperature (°C)	25, 30, 35, 40, 45
pH	5.0, 6.0, 7.0, 8.0, 9.0, 10.0, 11.0, 12.0
NaCl concentration (w/v %)	1, 5, 10, 15, 20, 30
Pb^2+^ concentration (mg/L)	500, 1000, 2000, 3000, 4000
Sampling time (h)	1, 3, 6, 12, 24, 48, 72

### 2.5 Instrumentation

The OD_600_ value of the bacterial liquid was determined at 600 nm using a UV visible spectrophotometer (752 NPlus, Shanghai Yidian, China). The Pb content in the solution was determined by ICP-OES (icap7000, ThermoFisher Scientific Inc iCAP PRO, United State). The 3D-EEM excitation wavelength is 200–600 nm, the emission spectrum wavelength was 200–600 nm, and the diffraction slit is 5 nm (F-7100, Hitachi, Japan). Organic acids are tested by high performance liquid chromatography (U3000, ThermoFisher Scientific Inc, United State) (Su et al., 2019). Refer to our previous research for the extraction and testing methods of EPS (Chen et al., 2021). Used the electrochemical workstation (CHI760D, CHInstrument Company, China) to conduct the electrochemical test of the Pb stress bacterial liquid by cyclic voltammetry (CV) (the working electrode is bacteria modified glassy carbon electrode, auxiliary electrode is platinum wire electrode, and the reference electrode is saturated calomel electrode). XRD (D8 Advance, Bruker AXS GMBH, Gemany, Cu Kα, λ= 1.540 60 Å; 40 kV; 40 mA; and 2θ rotation range of 10°–80°) and ATR-IR (Nicolet iS5 Fourier-transform infrared spectrometer, ThermoFisher Scientific Inc.) were used to determine the mineral composition and functional group characteristics in solid samples. The morphology and elements of solid samples were determined by SEM (Hitachi Regulus 8100, Japan) and EDX (INCA 300 Oxford, UK).

### 2.6 Statistical analysis

Repeat three times for each experiments. The means and standard deviations in each treatment were calculated and presented. The 16S rRNA sequences were compared by BLAST program, and then the phylogenetic tree was constructed by MEGA 7.0 software (Zhu et al., 2011; Biswas et al., 2018). Charting with Origin 2022 and Office 365.

## 3 Results and discussion

### 3.1 Screening and identification of CZ-B1

**FIGURE 1:**
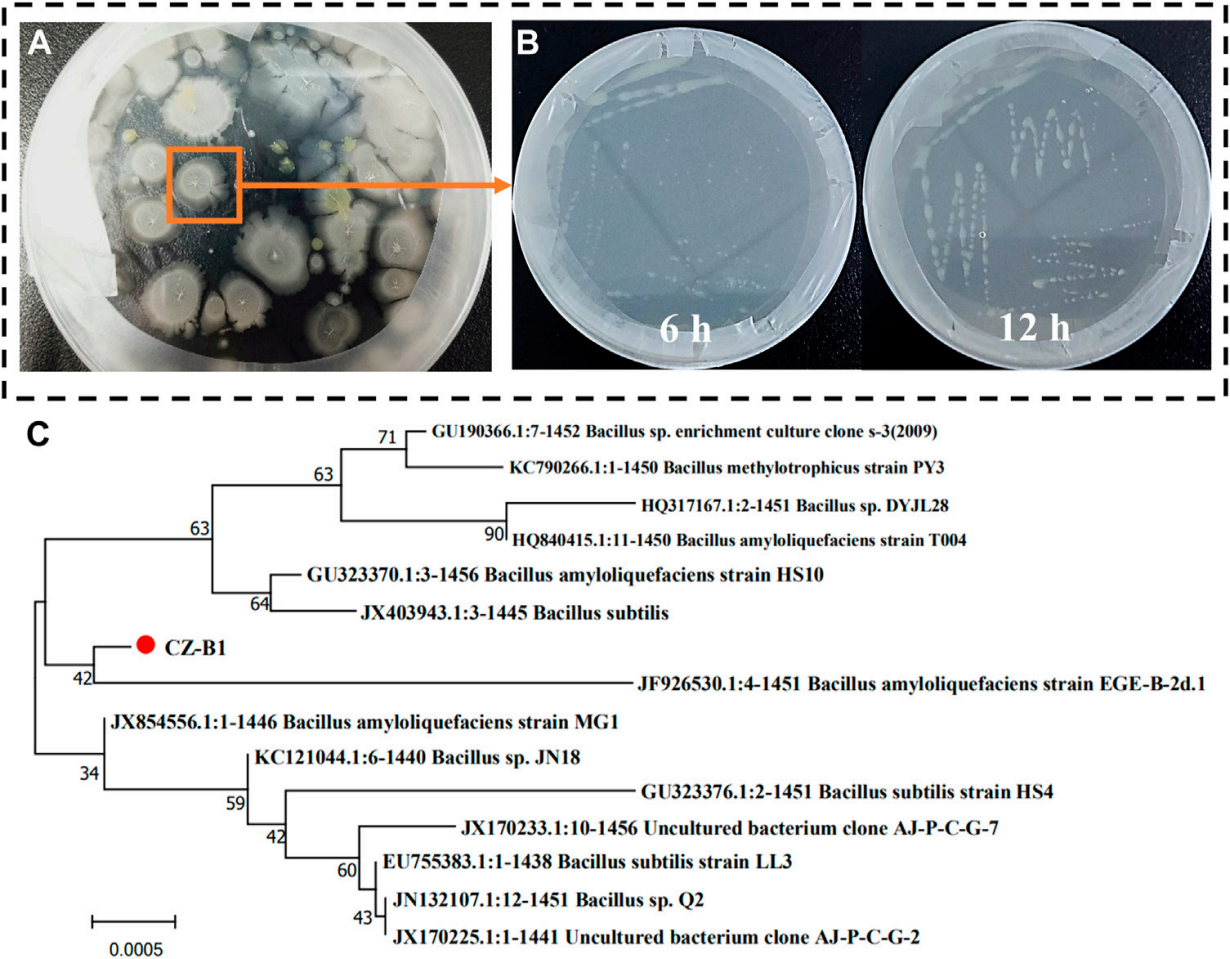
Preliminary screening **(A)**, growth status **(B)** and phylogenetic tree **(C)** based on 16 S rRNA sequence of CZ-B1.

CZ-B1 obtained by screening and purification from saline alkali soil and its state after 6 and 12 h culture are shown in Figures 1A, B The colony of CZ-B1 is milky white, opaque, rough and diffuse. In addition, CZ-B1 reached a good growth state within 12 h, which indicates that CZ-B1 grows rapidly and has strong environmental adaptability. The 16S rRNA amplification of strain CZ-B1 showed that its molecular weight was 1457 bp. The results showed that the 16S rRNA of strain CZ-B1 had the highest homology with *Bacillus velezensis* (GenBank registration number NR-116240.1) *Bacillus amylolyticus* (GenBank registration number NR-117946.1), and the sequence matching degree were both 99.10% (Supplementary Figure S1). Meanwhile, phylogenetic tree results showed that CZ-B1 and *B. amyloliquefaciens* EGE-B-2d. 1 (registration No. JF926530.1) were on the same branch, and the unit length of difference between sequences was 0.0005 (Figure 1C). Therefore, we confirmed CZ-B1 as a strain of *B. amyloliquefaciens*. The strain CZ-B1 was sent to China General Microbiology Preservation Center for preservation, with the preservation number of CGMCC11.19458.

### 3.2 Phosphorus solubilizing capacity of CZ-B1

**FIGURE 2:**
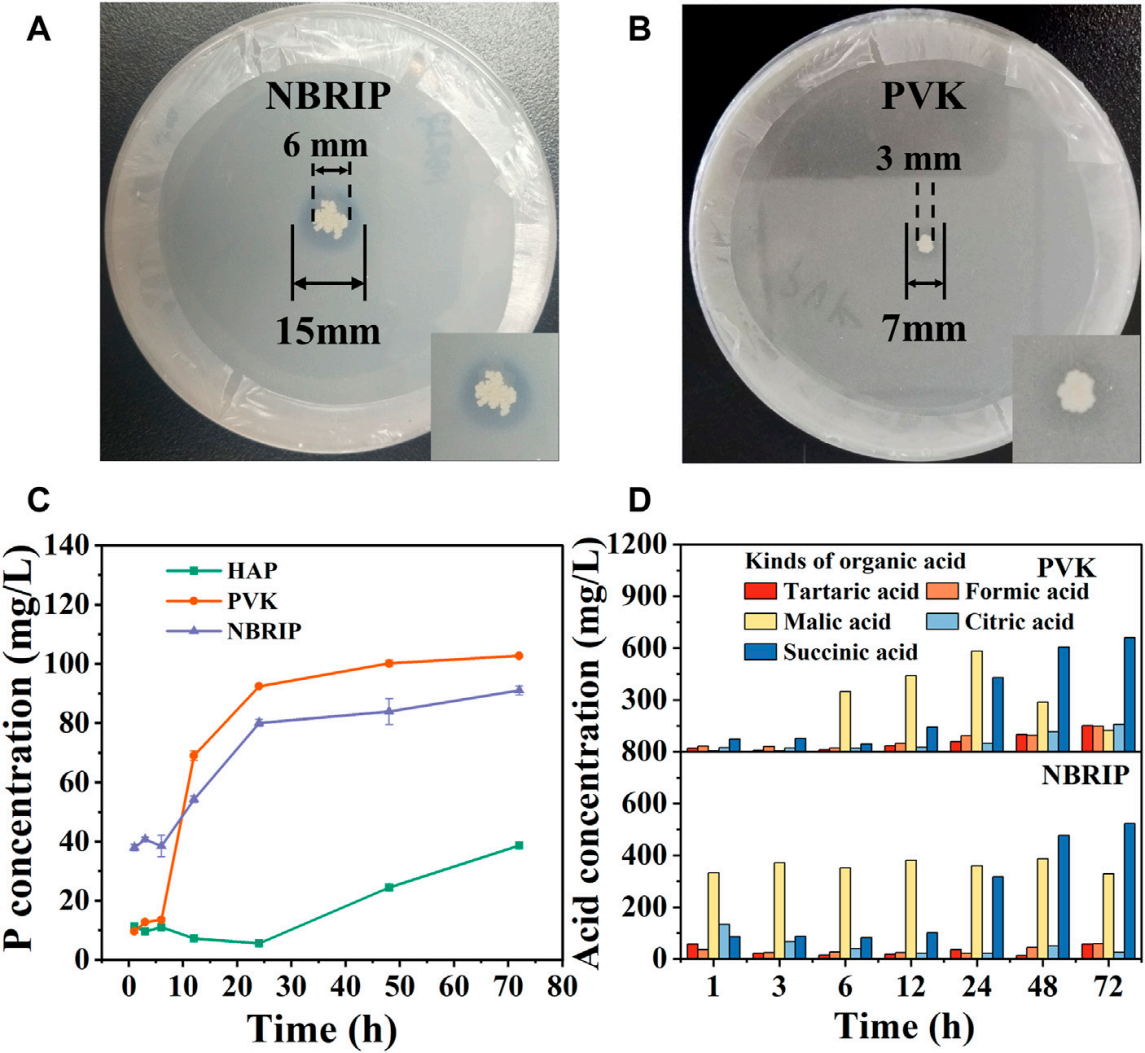
The ability of CZ-B1 to dissolve phosphorus and secrete organic acid. Transparent zone of phosphate solubilization on PVK **(A)** and NBRIP **(B)** for 7 days of CZ-B1, The soluble phosphorus **(C)** and organic acid **(D)** content of CZ-B1 in different media.

Figures 2A, B are divided into photos of CZ-B1 cultured on NBRIP solid medium and PVK solid medium for 7 days. The phosphoric acid solubility index (PSI) of CZ-B1 on NBRIP medium and PVK medium are 2.5 and 2.3 respectively, which can be used to determine that CZ-B1 was a phosphate solubilizing bacteria (Sarkar et al., 2012). In addition, the phosphorus content of liquid medium showed that the amount of dissolved phosphorus in CZ-B1 to HAP medium, PVK medium and NBRIP medium increased with time (Figure 2C). The phosphorus content in all three media increased slowly during the first 6 h, increased rapidly from 6 to 24 h, and then tended to be stable. Moreover, the trend of dissolved phosphorus of CZ-B1 in the three media at 72 h was PVK > NBRIP > HAP, and the dissolved phosphorus in PVK medium was 102.73 mg/L, 1.13 times of that in NBRIP medium.

In PVK medium, the concentration of formic acid, tartaric acid, citric acid and succinic acid increased with the increase of culture time, while the concentration of malic acid increased first and then decreased (Figure 2D). In NBRIP medium, the content of formic acid, tartaric acid, citric acid and malic acid fluctuated continuously, and the content of succinic acid increased with the increase of culture time. After 12 h, the tartaric acid, citric acid and succinic acid in PVK were gradually higher than those in NBRIP, and it was at this time that the phosphorus content in PVK medium was also gradually higher than that in NBRIP medium. Therefore, tartaric acid, citric acid and succinic acid may be the key for CZ-B1 to dissolve Ca_3_(PO_4_)_2_ in PVK medium. In addition, the amount of succinic acid secreted in the two media increased with the increase of culture time, which was in perfect agreement with the change trend of phosphorus content. Therefore, succinic acid may make a great contribution to the dissolution of Ca_3_(PO_4_)_2_ by CZ-B1.

### 3.3 Stress tolerance experiments with CZ-B1

**FIGURE 3:**
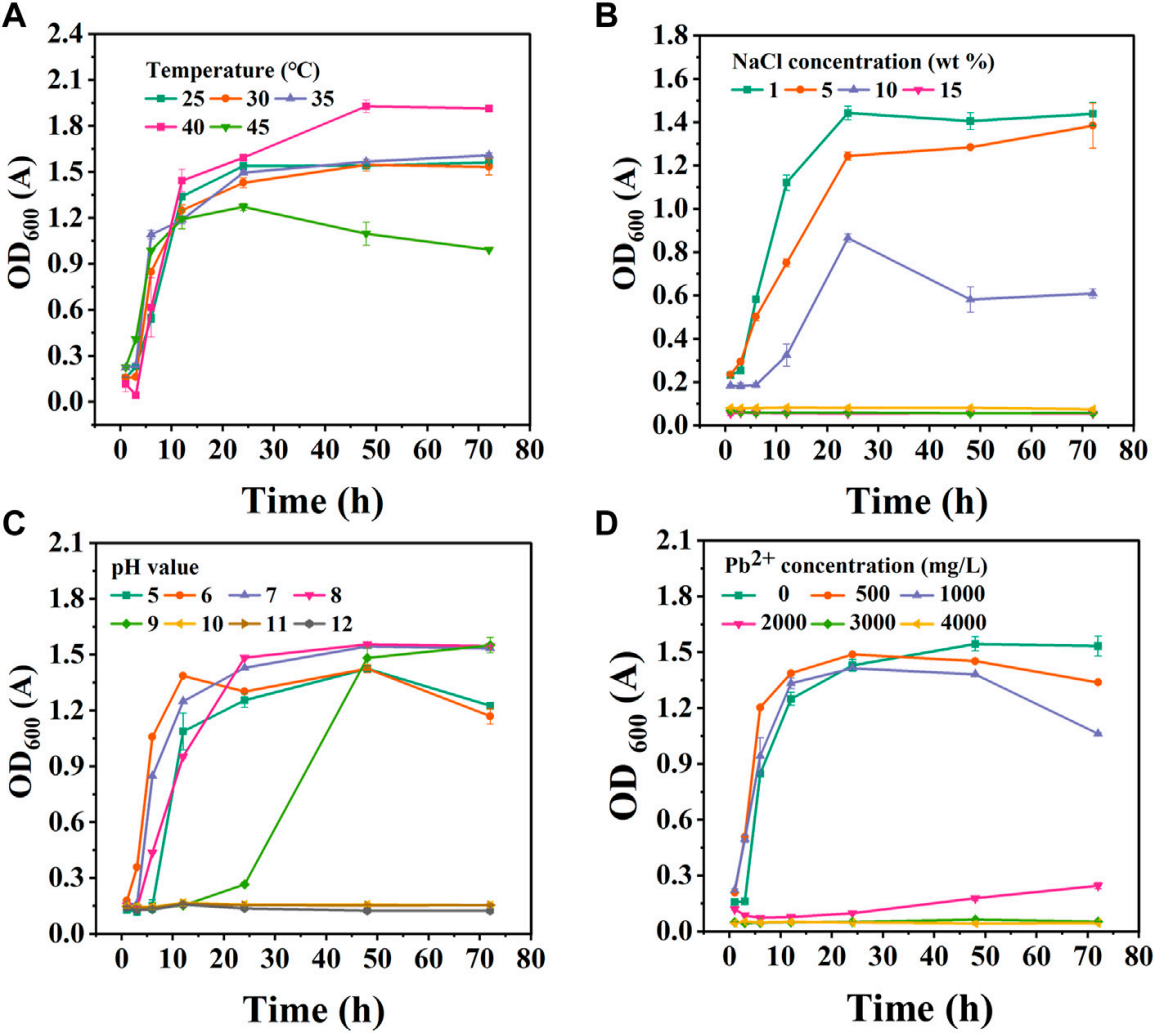
The growth status of CZ-B1 under different stress conditions. Temperature effect **(A)**, NaCl stress **(B)**, pH stress **(C)**, Pb^2+^ stress **(D)**.

The growth curves of CZ-B1 at different temperatures are shown in Figure 3A. The most suitable growth temperature of CZ-B1 was 40 C, and it can keep good survival in the temperature range of 25°C–45℃. At 25°C–40℃, the growth of CZ-B1 tends to be stable after 24 h. However, at 45℃, the growth of CZ-B1 gradually decreased after 24 h and the final OD_600_ value was only 51.8% of the OD_600_ value at 40℃. Nevertheless, CZ-B1 was able to maintain a high activity between 25°C–40°C, which indicates that CZ-B1 has a good adaptability to temperature.

In the salt stress experiment, the activity of the strain decreased with the increase of NaCl concentration (Figure 3B). When NaCl concentration reaches 15%, CZ-B1 cannot grow. Therefore, the maximum NaCl tolerance concentration of CZ-B1 is 100–150 g/L, which proves that CZ-B1 can grow in a high salinity environment. In addition, the growth of CZ-B1 showed rapid growth within 24 h under the stress of 1%–10% NaCl concentration. After 24 h, CZ-B1 growth remained stable under 1% NaCl stress, grew slowly under 5% NaCl stress, and appeared to decrease under 10% NaCl stress. Although the final OD_600_ of CZ-B1 under 10% NaCl stress was only 42.33% of that of 1% NaCl, CZ-B1 was still stable enough to survive. Therefore, CZ-B1 has good salt tolerance.

The OD_600_ value (72 h) of CZ-B1 was maximum at pH 7–9 (Figure 3C), and there was no significant difference among the three, which indicates that CZ-B1 is more suitable for survival under weak alkaline conditions. With the increase of pH (pH > 10), the growth of CZ-B1 was obviously restricted. Furthermore, although CZ-B1 can survive and maintain a high biomass at pH 9, it needs a longer lag period to adapt to pH stress (1–24 h). The growth rate of CZ-B1 was greatest at pH = 6 and was able to reach the stabilization phase faster. In addition, since CZ-B1 can also survive and maintain high activity in weakly acidic conditions (pH = 5–6), it may also be adapted to a less acidic environment. Therefore, the phosphorus solubilizing bacteria CZ-B1 can survive in a very wide pH range (pH 5–9) and the most suitable pH environment for CZ-B1 growth is 7-8.

The growth of CZ-B1 under different Pb^2+^ stresses was shown in Figure 3D. When the concentration of Pb^2+^ < 1000 mg/L, CZ-B1 shows a certain tolerance to Pb. The OD_600_ values (72 h) under 500 mg/L and 1000 mg/L Pb^2+^ stress were 87.2% and 69.2% of those without stress, respectively. However, when Pb^2+^ reached 2000 mg/L, the OD600 of CZ-B1 at 72 h decreased significantly and was only 16.0% of that without stress, which indicates that the growth of CZ-B1 was significantly inhibited. Although the initial stress effect of 2000 mg/L Pb^2+^ concentration on CZ-B1 was obvious, the OD_600_ of CZ-B1 gradually increased after 48 h. Therefore, CZ-B1 may need a longer time to adapt to the Pb^2+^ stress of 2000 mg/L. Although CZ-B1 cannot survive well at Pb^2+^ concentration higher than 3000 mg/L, its excellent tolerance made it have the potential for further domestication.

### 3.4 The mechanism of Pb remediation by CZ-B1

#### 3.4.1 Pb removal and organic acid

**FIGURE 4:**
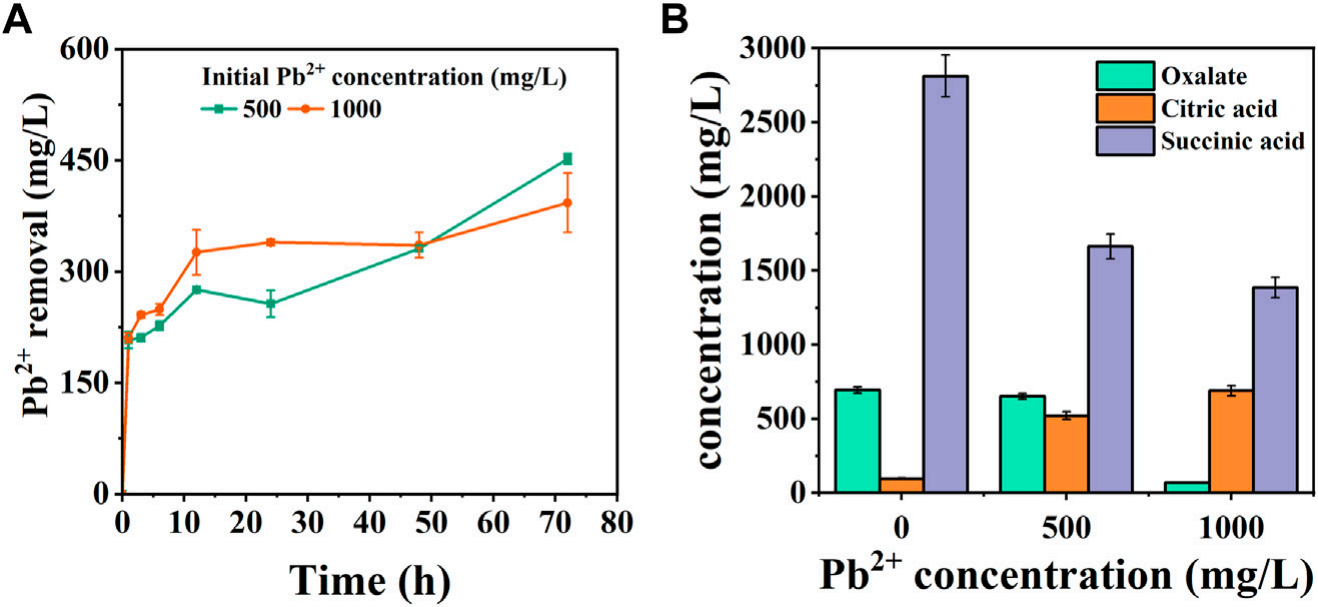
Pb^2+^ removal amount **(A)** and organic acid content **(B)** in solution under 500 mg/L and 1000 mg/L Pb^2+^ stress.

The strain CZ-B1 has a significant removal effect on Pb while resisting Pb stress (Figure 4A). The removal rate of Pb^2+^ in the treatment of 500 mg/L Pb^2+^ and 1000 mg/L Pb^2+^ by CZ-B1 at 72 h was 90.38% and 39.3% respectively. With the inverse ratio of Pb stress concentration to Pb^2+^ removal, this may be due to the fact that increased Pb^2+^ toxicity severely inhibits bacterial reproduction and growth functions, thus interfering with their Pb^2+^ adsorption. In addition, the removal of Pb^2+^ by CZ-B1 under 500 mg/L Pb^2+^ stress showed three stages of increase (0–12 h), decrease (12–24 h) and increase (24–72 h). Under the Pb^2+^ stress of 1000 mg/L, CZ-B1 was able to adapt to the Pb^2+^ stress within 24 h at the earliest and continued to perform its ecological restoration function.

Organic acid secretion is a key process for PSM to dissolve phosphorus sources in the environment (Su et al., 2019). Phosphate dissolved/released by PSM can form stable phosphate minerals with HMs ions (Xiao et al., 2021). In addition, the chelation of organic acids with HMs can reduce the toxicity of HMs, which is also a common way for microorganisms to resist HMs stress on their own (Que et al., 2020). Malic acid, citric acid, oxalic acid, etc. are small molecule organic acids usually secreted by PSM (Rawat et al., 2020; Sarmah and Sarma, 2022). In order to explore the mechanism of Pb^2+^ removal by CZ-B1, we determined six organic acids in the solution (Figure 2D). In LB medium without Pb^2+^ stress, oxalic acid, citric acid, malic acid and succinic acid, formic acid and tartaric acid were detected in the solution. This result was different from the oxalic acid content in the two inorganic media (Figure 2D), which could be attributed to the different stress of CZ-B1 itself to inorganic and organic phosphorus sources. Among them, the content of malic acid (6902.0 mg/L) > succinic acid (2812.7 mg/L) > citric acid (96.4 mg/L) > oxalic acid (68.7 mg/L) at 72 h (Supplementary Table S1). The maximum level of organic acid release from CZ-B1 in organic medium was significantly higher than that in inorganic medium.

Formic acid, malic acid and tartaric acid were not detected in solution under 500 mg/L-1000 mg/L Pb^2+^ stress, which suggesting that Pb stress may directly inhibit the secretion of CZ-B1 for the three acids. Meanwhile, oxalic and succinic acids in solution kept decreasing with increasing concentration of Pb stress, while citric acid gradually increased. The content of oxalic acid and succinic acid in the solution under 1000 mg/L Pb^2+^ stress was 10.5% and 83.3% of that under 500 mg/L Pb^2+^ stress, respectively, which indicated that the effect of Pb stress on oxalic acid content was greater than that on succinic acid content. Also, the increase of citric acid content in the solution (by 32.2%) with increasing Pb^2+^ concentration indicates that Pb^2+^ coercion stimulates the secretion of citric acid by CZ-B1. In addition, the decrease in oxalic and succinic acid content in solution could both be caused by slowed cellular metabolism and reduced activity or inactivation due to HMs stress (Babu et al., 2015). Studies have shown that environmental stress significantly inhibits the rate of tricarboxylic acid cycle in microorganisms, thereby reducing the secretion of succinate (an intermediate of the tricarboxylic acid cycle) (Guarnieri et al., 2017; Teng et al., 2019). In addition, some studies have shown that oxalic acid secreted by *Aspergillus niger* can form PbC_2_O_4_ precipitation with Pb^2+^ (Ceci et al., 2015; Feng et al., 2022). Therefore, the reduction of oxalic acid in solution may also be transferred to the precipitate in the form of Pb oxalate. On the other hand, the results of organic acid content per unit cell of CZ-B1 showed that the oxalic acid concentration of unit cell under 1000 mg/L Pb^2+^ stress was higher than that under 500 mg/L Pb^2+^ stress, which further confirmed that CZ-B1 could still release oxalic acid under Pb stress (Table 2).

**TABLE 2:** Unit concentration of different organic acids under 500 mg/L and 1000 mg/L Pb^2+^ stress.

Organic acid content per unit OD	Pb^2+^ concentration (mg/L)		
	0	500	1000
Oxalic acid (mg/L)	452.08	486.58	64.68
Citric acid (mg/L)	62.87	389.63	649.16
Succinic acid (mg/L)	1834.76	1242.77	1304.42

#### 3.4.1 3D-EEM result

**FIGURE 5:**
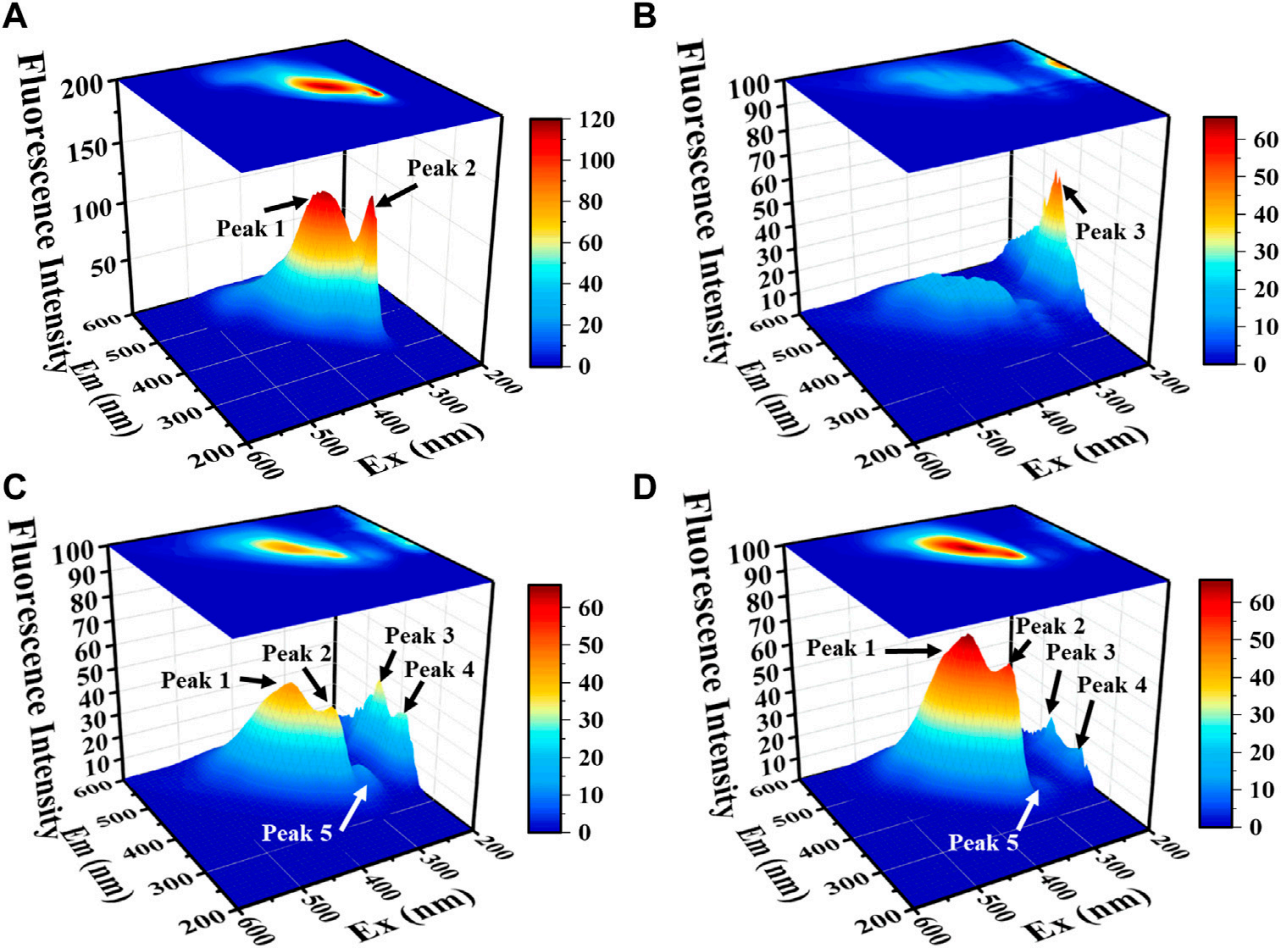
The 3D-EEM spectrum of organic matter in solution with LB medium **(A)**, no Pb^2+^ stress **(B)**, 500 mg/L Pb^2+^ **(C)** and 1000 mg/L Pb^2+^ **(D)** for 72 h.

Figure 5 there are five characteristic peaks were mainly present in the 3D-EEM of CZ-B1, among which peak 1 (Ex/Em 370/450) and peak 2 (Ex/Em 330/400) belonged to humic acid (Baker, 2001; Guo et al., 2014) and fulvic acid (Wang et al., 2009; Lin and Guo, 2020), which were mainly derived from the nutrients in LB. Peak 3 (Ex/Em 200/450) and peak 4 (Ex/Em 200/400) may originate from the secretion of CZ-B1. In addition, there is a small peak 5 at Ex/Em 280/380. Studies have shown that Ex/Em 280/380 may be a by-products of microorganisms (Chen et al., 2003) or tryptophan (Baker, 2001).

When the Pb^2+^ concentration increased from 500 mg/L to 1000 mg/L, the fluorescence intensity of peek 1 and peek 2 increased by 44.8% and 47.1%, respectively. Meanwhile, peak 3, peak 4, and peak 5 from microbial secretions were decreased by 50.1%, 52.6% and 60.0%, respectively. It can be concluded that the metabolic activity of the microorganisms decreased and the consumption of nutrients diminished with increasing Pb stress concentration and that microbial by-products such as tryptophan might be produced simultaneously under stress conditions. Tryptophan as a precursor of indoleacetic acid (Shahid et al., 2012), both of which possess the property of promoting phosphorus solubilization (Ji et al., 2012; Alemneh et al., 2021), which undoubtedly provides a gaining effect for Pb^2+^ passivation. Therefore, the increase of tryptophan can indirectly prove the enhanced bioimmobilization of Pb^2+^ by CZ-B1. This phenomenon also proves that under low-concentration stress, CZ-B1 are more inclined to use their own cells for Pb^2+^ fixation.

#### 3.4.2 XRD, ATR-IR and SEM result

**FIGURE 6:**
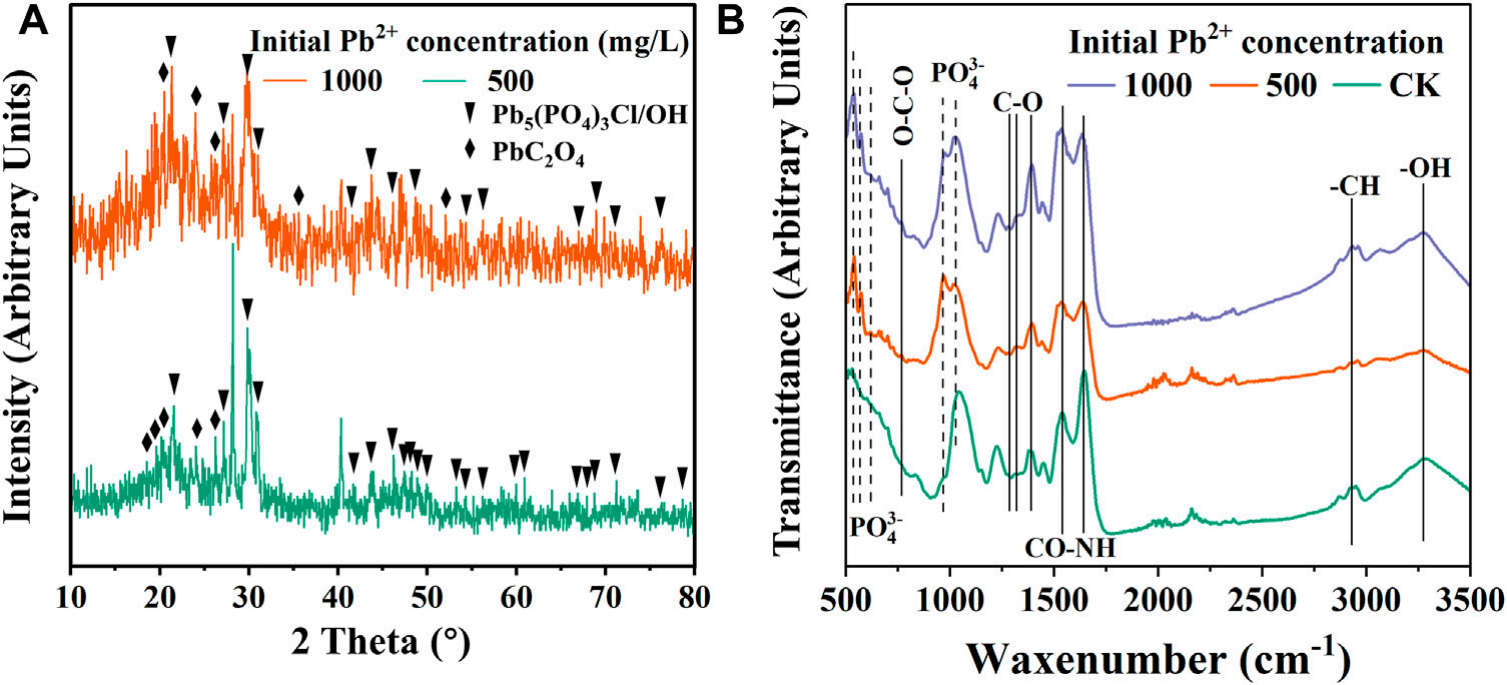
XRD **(A)** and ATR-IR **(B)** of precipitate under 500 mg/L Pb^2+^ and 1000 mg/L Pb^2+^ stress for 72 h.

XRD results showed that Pb-phosphate and Pb-oxalate were present in the sediment (Figure 6A). Many strong diffraction peaks were present at 29.8°–30.9°, 21.6°, 26.3°–27.2° and 43.9° in both concentrations of Pb^2+^ stress, which may be attributed to pyromorphite [Pb_5_(PO_4_)_3_Cl/OH] (Meng et al., 2020). Meanwhile, the diffraction peaks at 20.78°, 23.6°, 26.4° and 35.5° indicate the formation of PbC_2_O_4_ (Choi et al., 1994; Ma et al., 2016). Compared with 1000 mg/L Pb^2+^ stress, the pyromorphite peak signal of CZ-B1 under 500 mg/L Pb^2+^ stress was more significant. The formation of pyromorphite indicates that CZ-B1 can fix Pb2+ by releasing phosphorus. However, the Pb oxalate signal under 1000 mg/L Pb^2+^ stress is lower than that under 500 mg/L Pb^2+^ stress. Therefore, according to the oxalic acid content in solution under different Pb stress (Figure 4A), the decrease of oxalic acid under high concentration Pb stress may be more attributed to the decrease of cell number of CZ-B1 than to Pb oxalate precipitation. However, the formation of Pb phosphate and Pb oxalate can reduce the Pb concentration in the environment, which is also one of the main mechanisms of CZ-B1’s resistance to Pb stress.

ATR-IR results showed (Figure 4B) that the cell sediment under Pb stress increased the peak position related to PO_4_^3−^ at 565 cm^−1^, 603 cm^−1^, 965 cm^−1^ (Botto et al., 1997; Wei et al., 2021). In addition, the O-C-O stretching vibration peak of 782 cm^−1^ (Mancilla et al., 2009) and the C-O bond peak of 1289 cm^−1^, 1312 cm^−1^, 1386 cm^−1^ were enhanced (Mancilla et al., 2009; Salavati-Niasari et al., 2009). O-C-O and C-O may be related to Pb oxalate (Mancilla et al., 2009). This result further proves that there are Pb phosphate compounds and Pb oxalate compounds in the cell sediments under Pb stress. In addition, the peaks representing amides and vibration peaks of saturated aliphatic (-CH) substances were observed at 1533 cm^−1^, 1640 cm^−1^ and 2930 cm^−1^ (Doshi et al., 2007; Wang et al., 2020), indicating that the hydrocarbon functional groups and cellular proteins produced by CZ-B1 astress, which may be attributed topyromorphitelso participated in Pb^2+^ adsorption.

**FIGURE 7:**
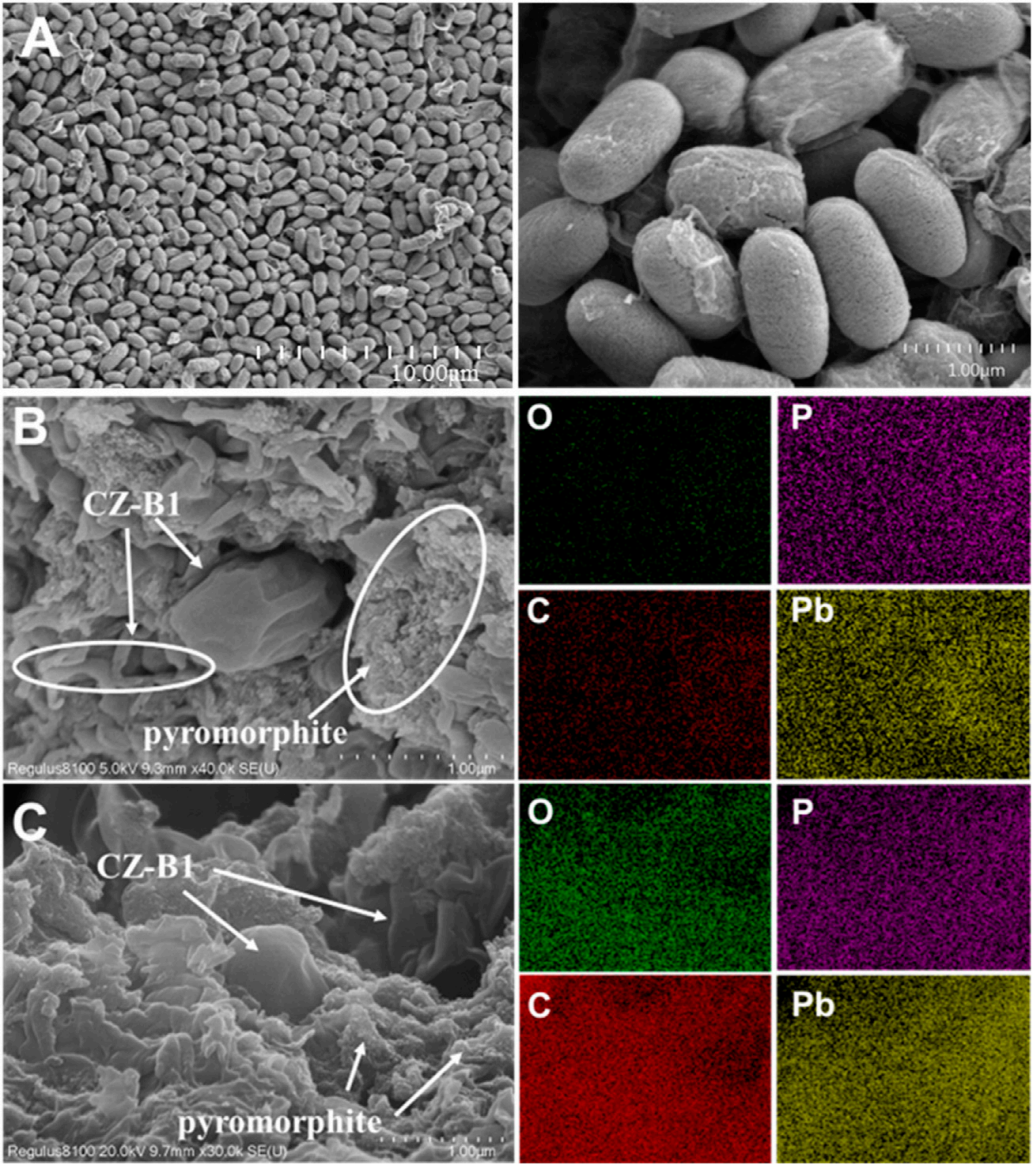
The SEM and EDX of precipitate under no Pb^2+^ stress **(A)**, 500 mg/L Pb^2+^ **(B)** and 1000 mg/L Pb^2+^ **(C)** for 72 h.

SEM images showed not only the presence of intact cells of CZ-B1 in the precipitate, but also the presence of obvious mineral grains (Figure 7). CZ-B1 cells are rod-shaped, about 2 μm, the surface is smooth (Figure 7A). Under Pb^2+^ stress, CZ-B1 cells appear deformation and shrinkage to varying degrees, and the cell morphology was significantly smaller, which indicates that Pb stress hinders the growth and development of CZ-B1 (Figures 7B, C). In addition, the individual CZ-B1 cell becomes smaller under the stress of 500 mg/L Pb^2+^, but the cell surface was smooth. The deformation of CZ-B1 under 1000 mg/L Pb^2+^ stress was more serious than that under 500 mg/L Pb^2+^ stress, and even broken cell remains were present in the precipitate (Figure 7C). In addition, EDX spectroscopy found that there were sharp particles with highly overlapped P and Pb signals around CZ-B1 cells, which may form pyromorphite. However, the pyromorphite signal was stronger under 1000 mg/L Pb^2+^, so more Pb phosphate mineral precipitation may be produced under the stress of high concentration of Pb. Combined with the OD values it can be confirmed that under high Pb^2+^ stress, the solubilization and utilization of P by CZ-B1 may be preferentially supplied to mineral precipitation rather than used by itself. The resistance mechanism of microorganisms under high stress may be dominant over the reproduction mechanism.

#### 3.4.3 Electrochemical analysis results

**FIGURE 8:**
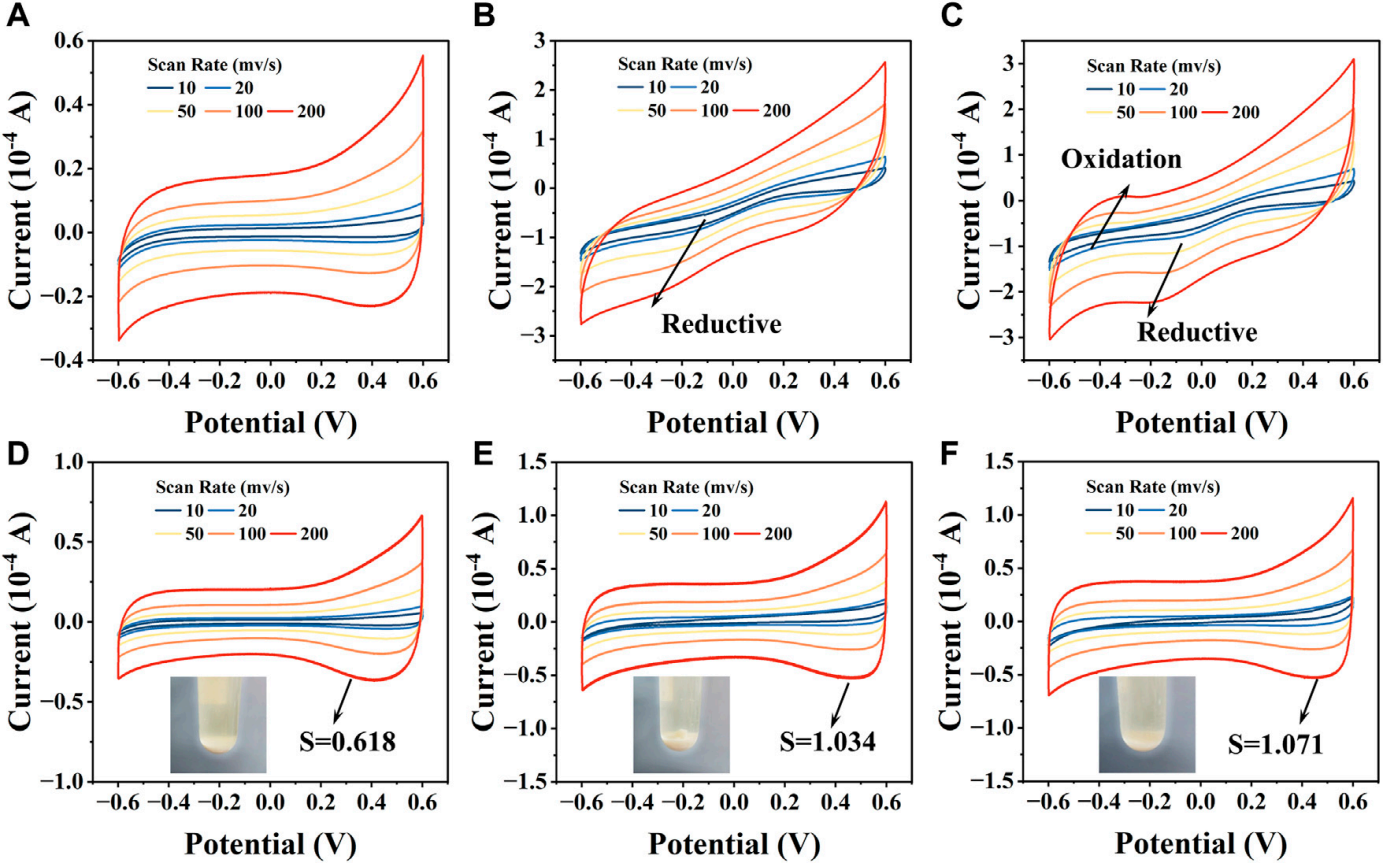
The CV curves of cell precipitates and EPS under no Pb^2+^ stress **(A, D)**, 500 Pb^2+^ **(B, E)**, and 1000 Pb^2+^ **(C, F)** stress.

The CV curves of CZ-B1 cell precipitates showed no significant redox peak positions in the absence of stress (Figure 8A). The CV curves under 500 mg/L Pb^2+^ stress showed significant reduction peaks between −0.4 V and −0.2 V, but not significant oxidation peaks (Fig. B). When Pb^2+^ stress was increased to 1000 mg/L, the reduction peak position of the CV curve shifted to between −0.4 V and 0.0 V, and a clear oxidation peak appeared around −0.4 V (Figure 8C). The generation and loading of minerals can change the electron transfer capacity of cell (Zhang et al., 2021), so the shift in the redox peak position of the CV curve may originate from the Pb minerals generated by CZ-B1. In addition, there were no significant oxidation or reduction peaks on the CV curves of the three different treatments of EPS (Figure 8D, E, F). However, the EPS peak area of CZ-B1 increased significantly by 67.15%–73.16% under Pb stress, which suggests that EPS may assist CZ-B1 in the removal of Pb^2+^ through adsorption. In addition, the area of CV closure increased continuously with increasing Pb stress concentration. The enrichment of Pb^2+^ by EPS was beneficial to increase the extracellular electron transfer rate and accelerate the mineralization response of cells to Pb^2+^, which is consistent with Figure 8C and XRD results. Compared to the cells, the main change in the CV curve of EPS was the peak area rather than the redox peak position. Therefore, we speculate that EPS mainly acts as an enriched adsorption for Pb^2+^ rather than generating Pb minerals on it. It is worth mentioning that the mineralization and precipitation of cells to Pb^2+^ were improved precisely because Pb^2+^ was adsorbed around CZ-B1 cells by EPS.

In summary, the resistance and repair mechanism of CZ-B1 to Pb stress may be mainly from three aspects: 1) CZ-B1 can combine with Pb^2+^ to form phosphate, succinic acid and oxalate minerals *via* secreted citric, succinic and oxalic acids. 2) CZ-B1 can secrete tryptophan and other microbial products for complexation to enhance the absorption of Pb^2+^, thus weakening the stress of Pb^2+^ on itself. 3) CZ-B1 adsorbs and enriches Pb^2+^ through pericellular and surface EPS to increase the extracellular electron transfer rate, which can increase the reaction rate of CZ-B1 with Pb^2+^ mineralization and promote mineral precipitation.

## 4 Conclusion

In this study, a phosphorus solubilizing bacteria strain CZ-B1 with Pb and salt tolerance was successfully screened. CZ-B1 is highly adaptable to temperature and can survive from 25°C to 45°C (the optimum temperature is about 40°C). CZ-B1 can survive in alkaline environment with pH < 10 and high salt environment with NaCl concentration <15%. In addition, the solubility of CZ-B1 for insoluble phosphorus source Ca_3_(PO_4_)_2_ could reach 102.8 mg/L within 72 h. Its efficient solubility for insoluble phosphorus source was attributed to succinic acid. The removal rate of 500 mg/L Pb^2+^ by CZ-B1 in liquid LB medium can reach 90.38%. With the increase of Pb concentration, the number of CZ-B1 cells decreased significantly, which greatly affected its cell adsorption function. However, the release of organic acids by CZ-B1 under Pb stress was very different from that under no Pb stress. Therefore, in addition to cell adsorption, the resistance/repair mechanism of CZ-B1 to Pb stress may be related to organic acids. The cells mainly secrete oxalic acid, citric acid and succinic acid under 500–1000 mg/L Pb^2+^ stress. Among them, citric acid increased with the increase of Pb^2+^ stress concentration, which may be the main mechanism of CZ-B1 resistance to Pb^2+^ stress. In addition, XRD and SEM results indicate that CZ-B1 reduces Pb^2+^ toxicity mainly through the formation of a large amount of pyromorphite minerals and a small amount of Pb oxalate precipitation. The formation of phosphate and oxalate benefited from the release of succinic acid and oxalic acid from CZ-B1, respectively. More than that, CV results showed that Pb minerals were mainly concentrated on CZ-B1 cells and Pb stress could affect the electron transfer process of CZ-B1. Therefore, Pb^2+^ may undergo mineralization reactions on or inside the cell after EPS enrichment.

## Data Availability

The original contributions presented in the study are included in the article/Supplementary Materials, further inquiries can be directed to the corresponding authors.
