# Supplementary material for: Isolation and screening of phosphorus solubilizing bacteria from saline alkali soil and their potential for Pb pollution remediation
Source: Front Bioeng Biotechnol. 2023 Feb 6;11:1134310. doi: 10.3389/fbioe.2023.1134310 (PMC9939700; doi:10.3389/fbioe.2023.1134310)

**Supplementary Information**

Isolation and screening of phosphorus solubilizing bacteria from saline alkali soil and their potential for Pb pollution remediation

Chaonan Zhang^1,#^, Haoming Chen^1,#*^, Yao Dai^1,^, Yan Chen, Yuxin Tian, Zongli Huo^2,*^

*^1^ School of Environmental and Biological Engineering,* *Nanjing University of Science and Technology, Nanjing 210094, China.*

*^2^ Jiangsu Provincial Center for Disease Control and Prevention, Nanjing 210094, China*

*^#^ These authors have contributed equally to this work*

**Corresponding to:**

**Zongli Huo**

Jiangsu Provincial Center for Disease Control and Prevention,

No.172 Jiangsu Road, Jiangsu, Nanjing, 210009, PR China.

Tel. +86(25) 83759917; Fax: +86(25) 83759917

E-mail: huozong123@163.com

**Haoming Chen**

School of Environmental and Biological Engineering,

Nanjing University of Science and Technology,

Nanjing, Jiangsu, 210094, China.

Tel. +86(25) 84315086; Fax: +86(25) 84315086

E-mail: [chenhaoming89@njau.edu.cn](mailto:lizhen@njau.edu.cn)

**Table S1. The organic acid content of CZ-B1 in LB medium with no Pb^2+^ stress for 72 h.**

| Organic Acid Concentration (mg/L) | Time (h) | | | | | | |
| --- | --- | --- | --- | --- | --- | --- | --- |
|  | 1 | 3 | 6 | 12 | 24 | 48 | 72 |
| Oxalic acid | 1551.333 | 475.119 | 672.578 | 664.554 | 126.092 |  | 693.035 |
| Tartaric acid | 46.697 | 128.874 |  |  |  |  |  |
| Formic acid |  |  |  |  |  |  |  |
| Malic acid | 37180.646 | 5975.663 | 1054.597 | 4527.728 | 5733.613 | 36523.793 | 6902.007 |
| Citric acid | 507.066 |  |  |  | 617.021 |  | 96.379 |
| Succinic acid |  | 36.305 | 228.629 | 772.547 | 1214.620 | 1770.747 | 2812.683 |

**Figure S1.** **The comparison results of 16S rRNA sequences of CZ-B1 in NCBI database.**


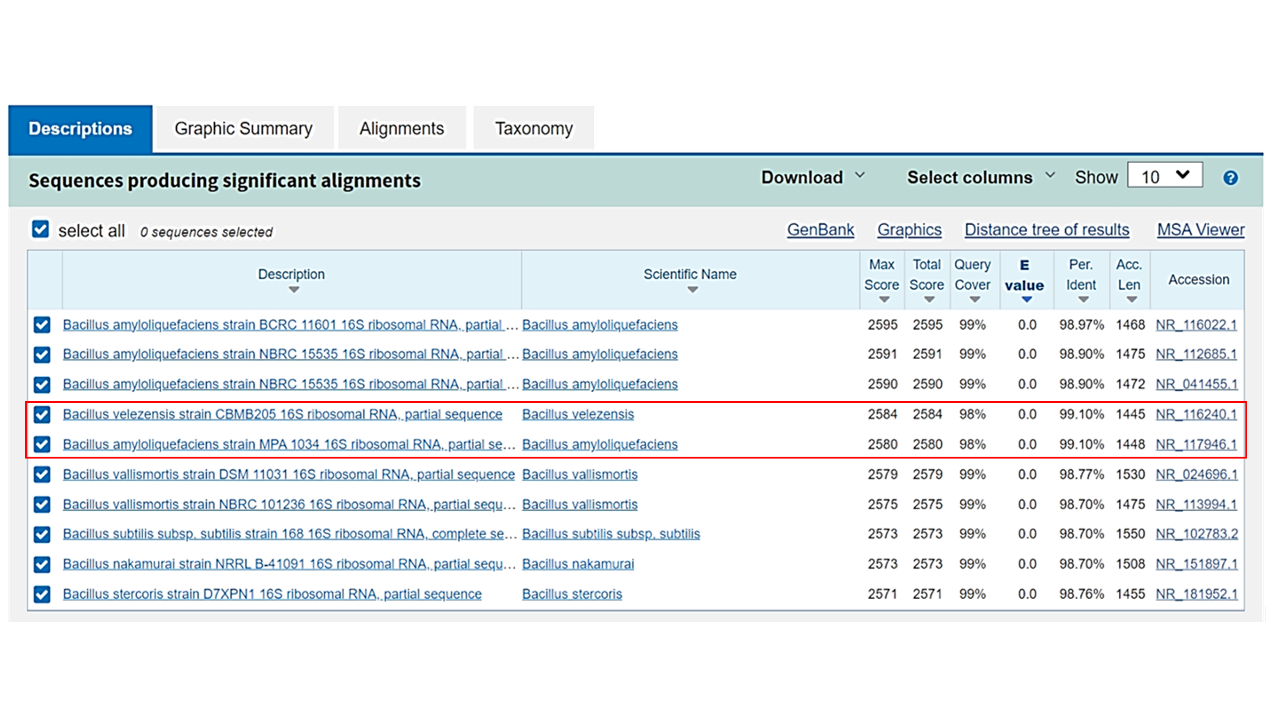

Supplement: Supplementary file 1 [file DataSheet1.docx]
